# A Randomized Controlled Trial Evaluating the Effectiveness of a Short Video-Based Educational Program for Improving Mental Health Literacy Among Schoolteachers

**DOI:** 10.3389/fpsyt.2021.596293

**Published:** 2021-02-26

**Authors:** Junya Ueda, Satoshi Yamaguchi, Yasuhiro Matsuda, Kosuke Okazaki, Tsubasa Morimoto, Seiya Matsukuma, Tsukasa Sasaki, Toshifumi Kishimoto

**Affiliations:** ^1^Department of Psychiatry, Nara Medical University School of Medicine, Nara, Japan; ^2^Department of Physical and Health Education, Graduate School of Education, University of Tokyo, Tokyo, Japan

**Keywords:** mental health literacy, stigma, schoolteacher education, mental health, video education, randomized controlled trial

## Abstract

**Background:** Mental illness-related stigma represents a barrier to seeking and receiving appropriate mental health care. Mental health literacy (MHL) can improve mental health knowledge, decrease stigmatizing attitudes, and enhance help-seeking behavior. Starting from 2022, mental illness-related education is due to be introduced in high schools in Japan. For this current situation, we conducted a parallel group, randomized controlled trial to examine the effectiveness of MHL educational program for teachers.

**Methods:** The educational program described in this study comprised a 50-min video lesson designed to improve teachers' MHL. All participants were schoolteachers and were assigned either to an educational group or a waitlist control group. The assessment was conducted for both groups twice: first at baseline and then at 1-h post-intervention. The outcome measures for this trial were changes in knowledge, attitudes, and intended behaviors.

**Results:** The educational group showed a greater improvement in knowledge regarding mental health than did the control group. The program was not effective for decreasing stigma toward mental illness. However, the educational group showed an increased intention to assist students with depression.

**Limitations:** No long-term follow-up was implemented, which means the persistence of the educational program's effect could not be determined. Further, we could not report whether the program induced a change in teachers' behaviors regarding providing support for their students.

**Conclusions:** The short video-based MHL educational program could improve schoolteachers' MHL and increase their intention to assist students. These findings can help in the development of similar educational programs in countries/regions experiencing similar issues regarding mental health.

## Introduction

Mental disorders are the most important cause of disability for 20–50-year-olds and account for the increase in total disease burden during early adulthood (1). Many people develop mental illness as teenagers [(2), p. 593−602]; however, a large number of young people with mental disorders tend to not seek help or support [(3), p. 113]. As a result, young people often lack adequate support and treatment, resulting in severe impairment of their social functions [(4), p. 1026–32]. In Japan, the number of young people with mental illness is also increasing (5); the country's Ministry of Health, Labor and Welfare reports that the rate of suicides as a result of mental illnesses such as depression and schizophrenia remain high (6).

Goffman (7) defined stigma regarding mental illness as “a trait that is deeply discrediting that reduces the bearer from a whole to a tainted, discounted one.” A more recent definition describes such stigma as negative attitudes and beliefs that motivate individuals to fear, reject, avoid, and discriminate against people with mental illness [(8), p. 765–76]. That is, stigma relates to ignorance, prejudice, and discrimination. Stigma can lead to reduced autonomy and self-efficacy, as well as segregation [(9), p. 907–22; (10), p. 619–25]. Such mental illness-related stigma, along with a lack of associated knowledge, can create a barrier that prevents youths from seeking help and accessing treatment services [(11), p. 11–27]. Therefore, dispelling stigma is very important for their mental health.

Mental health literacy (MHL) is designed to reduce stigma [(12), p. 154–58] via five components: (1) knowledge regarding means of preventing mental disorders, (2) ability to recognize when a disorder is developing, (3) knowledge of help-seeking options and available treatments, (4) knowledge of effective self-help strategies for milder problems, and (5) first-aid skills to support others who are developing mental disorders or who are experiencing a mental-health crisis [(13), p. 231–43]. Providing education regarding mental disorders and associated treatment methods can result in improving knowledge and reducing stigma. Indeed, the World Health Organization recommends that mental health promotion activities be implemented in schools (14).

Although MHL programs have already been implemented in schools in other countries [(15), p. 11–27], no such program has yet been included in the school curricula developed by Japan's Ministry of Education, and no information regarding mental health has been included in Japanese school textbooks for over 40 years [(16), p. 941–48]. However, because the number of suicides among young people with mental disorders has not decreased, the Ministry of Health, Labor and Welfare's 2017 suicide-prevention guidelines included “further promoting suicide prevention for children and adolescents” and “provision of education regarding how to cope in difficult situations and when experiencing severe psychological burden (i.e., how to send an SOS).” In addition, the course of study due to be implemented in high schools from 2022 includes the provision in health and physical-education classes of active content regarding mental-health education (17).

The aforementioned developments indicate that the need for MHL education in schools will soon increase. However, most teachers do not have sufficient knowledge regarding mental illnesses and have no experience of teaching such a discipline to students [(18), p. 452–73]. In addition, Rinke et al. (19) reported a global lack of experience and training for supporting children's mental health needs. Both a lack of knowledge regarding mental health and the stigma attached to mental illness impair teachers' ability to identify children who are experiencing mental disorders, as well as their ability to educate and relate to those children [(20), p. 61–8]. As a result, it is clearly necessary to provide MHL education to teachers before they begin to provide such education to students.

Research on MHL in Japan has been conducted sporadically. Only one study specifically focused on developing a program for schoolteachers [(21), p. 358–66]; however, this was a before–after comparative study, and was not a randomized controlled trial (RCT). Consequently, as there was no existing standardized educational MHL tool supported by scientific evidence Yamaguchi et al. [(22), p. 14–25] developed MHL education for teachers that featured an original 50-min video (DVD). In Yamaguchi et al. [(22), p. 14–25] pilot study, the authors reported that teachers' knowledge about mental disorders was improved in the single group before-and-after comparison.

Given the paucity of evidence of effective MHL education programs for teachers in Japan, and the encouraging results observed in Yamaguchi et al. [(22), p. 14–25] pilot study, it is necessary to assess the efficacy of such a program on a larger scale.

Therefore, in this study, we conducted an RCT as an extension of the pilot study to research whether schoolteachers' knowledge of and stigma regarding mental illness could be improved through the MHL intervention featuring this DVD program.

## Materials and Methods

### Trial Designs

This study comprised a two-arm, parallel-group, non-blinded RCT. Reporting of the results of this study is in accordance with the CONSORT 2010 Statement [(23), p. 100–7].

Teachers who consented to participate were individually and randomly assigned either to the educational group (which received the intervention) or the control group (which simply waited for the educational group to complete the program), for which a 1:1 ratio was used. Assignments were performed using computer-generated random numbers. Randomization was stratified by gender and age (<37, ≥ 37 years). The intervention delivery team was not involved in the randomization procedure. It was not possible for participants to be blind to intervention status. However, other staff members who assisted with data collection, and data input and statistical support were blinded to the group assignments.

### Participants

This study targeted schoolteachers. Participants were recruited between April 2018 and May 2019. Exclusion criteria included refusing to provide informed consent and withdrawal of consent. All teachers in the targeted schools were approached, irrespective of their gender. All teachers were allowed to participate, regardless of the type of school to which they belonged.

### Interventions

The intervention comprised watching a 50-min anime film on DVD. The short video [(24), p. 14–25] was developed by experts in child and adolescent psychiatry and early education, who were members of the Department of Physical and Health Education, Graduate School of Education, University of Tokyo.

The short educational video provided information regarding the epidemiology of mental disorders, the most prevalent psychiatric problems among children and adolescents, general descriptions and examples of the clinical symptoms of mental disorders (depression, panic disorder, schizophrenia, eating disorders, alcohol use, etc.), the importance and necessity of seeking help, means of responding to students' attempts to obtain help, and means of securing cooperation between schools and medical institutions.

### Outcomes

The participants completed self-report questionnaires regarding their socio-demographic characteristics. Before and after the program, they completed questionnaires regarding their mental health-related knowledge, as well as the Japanese version of the Reported and Intended Behavior Scale (RIBS-J), which was used to determine their intentions to provide help to students with depressive symptoms.

### Questionnaires

The assessment questionnaire comprised five domains (A–E). It was developed by combining self-developed items (domains A, B, and D) and items from existing measures (domains C and E). The five domains of the questionnaire were structured as follows:

(A) General knowledge about mental health/illnesses. The first part of the questionnaire comprised 19 questions regarding general knowledge about mental health/illnesses (Table 1). The possible answers to these questions were: “True,” “False,” or “I don't know.” Correct answers were scored 1 (otherwise scored 0) and the scores were summed. The total score for this domain (0–19 points) represented the participants' knowledge regarding mental health/illnesses, which was the primary outcome of this study.

**Table 1:** General knowledge about mental health/illnesses.

1. The incidence of most mental illnesses sharply increases in adolescence.
2. About one in every 20 people will experience a mental illness.
3.Staying up late and lack of sleep influence the development of and worsen mental health/illnesses.
4. Duration of treatment for depression and schizophrenia is about half a year on average.
5. People with mental illnesses may only have somatic symptoms, including headaches, abdominal pain, and nausea.
6. When depressed mood, if decreased motivation and diminished interest continue over time, it may be major depression.
7. In depression, both lack of sleep or insomnia, and oversleeping are possible.
8. People with mental illnesses may have difficulty riding vehicles (i.e., taking public transportation), leading to difficulties in attending school.
9. Auditory hallucinations and delusions of being persecuted can be treated by talking.
10. More than 10% of people will experience depression.
11. Approximately 1% of people will experience schizophrenia.
12. Asking about suicidal ideation should be avoided, because it can lead to suicide attempts.
13. Students should return to school after treatment for their mental illness has been completed.
14. When you cannot sleep, drinking alcohol can help you sleep better.
15. Drinking alcohol worsens anxiety and depression.
16. People with bipolar disorder are mostly identified when they are depressed.
17. Due to a mental illness, people may be unable to talk to others due to worry/nervousness.
18. In high school students, 7-h of sleep is best to decrease the risk of depression.
19. When you view bright lights late at night, you will have difficulty falling asleep.

(B) Measurement of the participants' ability to recognize specific mental disorders (depression, panic disorder, and schizophrenia). In this domain, the teachers were given three case vignettes describing three adolescent students with symptoms of depression, schizophrenia, and panic disorder. Having read each vignette, teachers were asked to give the name of the illness each student was experiencing. The answer was selected from 6 choices: “no illness,” “depression,” schizophrenia,” “panic disorder,” “social phobia,” and “I don't know” (Table 2).

**Table 2:** Vignettes for depression, schizophrenia, and panic disorder.

Q1	Student A goes to the health care room in the school, reporting having a headache and stomachache, and feeling tired. Student A has trouble sleeping, doesn't feel like eating, doesn't have fun watching his/her favorite TV program, and can't keep his/her mind on his/her studies. Student A is often late for school these days.
Q2	Student B appears to have trouble concentrating in class, compared to before. Student B covers his/her ears during break time. When asked, Student B says, “I feel that someone is always spying on me. People in class are always saying bad things about me/talking about me behind my back. When strangers pass by, I feel like they are also saying bad things about me. I feel nervous and concerned about noises and voices in the surroundings.”
Q3	In the bus on the way to school, Student C sometimes suddenly feels his/her heart pounding and has difficulty in breathing. When this happens, cold sweats and trembling do not stop, and Student C feels scared that he/she will suddenly die. Due to fear of this happening again, Student C became unable to take the bus.

(C) Attitudes toward students with depressive symptoms. Items 1–3 from the Depression Stigma Scale [DSS; [(25), p. 342–49]] with the construct “weak-not-sick” (item 1: “People with depression could snap out of it if they wanted;” item 2: “Depression is a sign of personal weakness;” and item 3: “Depression is not a real medical illness”) was used. One of the DSS items “It is best to avoid people with depression so you don't become depressed yourself” was excluded; the item may be inappropriate for school teachers. The items were scored using a five-point Likert scale ranging from 1 (“strongly agree”) to 5 (“strongly disagree”). The total scores could range from 3 to 15, with lower scores indicating greater stigma.

(D) Measurement of the participants' intentions to help students with depressive symptoms. In this domain, the teachers were asked, “When you encounter students like student A, you will consult with.” and were presented with a list of 10 possible people to consult (targets to consult). The 10 targets to consult could be classified as “in the school” or “outside the school.” Answers were provided using a six-point Likert scale ranging from 1 (“strongly disagree”) to 6 (“strongly agree”) (Table 3).

**Table 3:** Intentions to help student with depressive symptoms.

**When you encounter to students like student A, you will consult with**.
1. Family of the student
2. Your boss
3. Health care teacher
4. Colleague teacher
5. School counselor
6. Social worker
7. School doctor
8. Experts out of school
9. The student himself
10. Friends of the student

(E) Behavior regarding mental health-related stigma (the RIBS-J). The RIBS [(26), p. 263–71] measures behaviors relating to stigma regarding mental health. It can be administered to participants from the general public in conjunction with attitude- and behavior-related measures. The RIBS-J, developed by Yamaguchi et al. (27), has good internal consistency, and reasonable test-retest reliability and construct validity, similar to the original version. Thus, it can be considered an appropriate and psychometrically robust scale for assessing behavior regarding mental health-related stigma. The RIBS-J comprises two subscales, both of which contain four items. The first subscale (the “past domain”), which assesses “reported behavior,” includes four statements relating to past or present contact with people with mental health problems. In this domain, “yes” answers are awarded a score of one, and “no” or “don't know” answers are scored zero. The second subscale (the “future domain”) comprises four questions, which assesses the attitude toward people with mental health problems in the future. In this domain, scores are provided using a five-point Likert scale ranging from 1 (“strongly agree”) to 5 (“strongly disagree”). The total score for each participant is calculated by adding the response values; “don't know” is coded 3, indicating neutrality.

In the present study, Cronbach's alpha values for each domain were 0.77, 0.73, 0.82, 0.81, and 0.78 for A, B, C, D, and E, respectively.

### Statistical Analysis

Baseline significance tests comparing the groups of participants were conducted, with independent *t*-tests performed for age, years of work as teachers, and RIBS past domain, and chi-square tests for sex, experiences of attending a seminar about mental health, and experiences of involvement with people with mental illness.

Mixed-effects models, i.e., linear mixed models (LMM) and logistic regression mixed models (LRMM), were used for analyses of continuous and dichotomous outcome variables. Mixed models are appropriate for the analysis of longitudinal data and nested data. They are also robust against missing data in outcome variables, provided that the variables are missing at random [(28), p. 440–59]. In the present study, all participants who completed the questionnaire at pre-test were included in the analyses.

LMM were used for continuous variables (the total score for the domain A, C, D, and E). Equations for the LMM were as follows:

Level 1:

(1) $$\begin{array}{r} Y_{ti\;}=\;\beta_{0i}+\;\beta_{1i}\left(post-test\right)+\;r_{ti}\# \end{array}$$

Level 2:

(2) $$\begin{array}{r} \beta_{0i}=\gamma_{00}+\;\gamma_{01}\left(group\right)+\;\mu_{0i}\# \end{array}$$

(3) $$\begin{array}{r} \beta_{1i}=\;\gamma_{10}+\;\gamma_{11}\left(group\right)\# \end{array}$$

The dependent variable (Y_*ti*_) was the total score of the domains A, C, D, and E (the future domain). The measurement occasions (time) were nested within teachers. Thus, the effects of time and group were estimated at Level 1 and Level 2, respectively. The subscripts *t* and *i* refer to time and individual, respectively. At Level 1 [equation (1)], the intercept and the effects of post-test were represented by β (unstandardized regression coefficients). The residuals are represented by r_*ti*_. At Level 2 [equation (2) – (3)], the model included the effects of group [γ_01_ for the intercept (2)]. The interaction between post-test and group was represented by γ_11_. The interaction shows the effects of the video program in the education group compared to the control group. The significance of the coefficient of interaction determined the effect of the intervention. Residuals for the Level 2 equations are indicated by μ_0i_. Effect sizes (d) were calculated from the mean difference between pre-test and post-test divided by pooled standard deviation (SD_pooled_) from the intervention and control groups at pre-test [(29), p. 43–53].

LRMMs were used for the outcomes of domain B where the answers to the questions were dichotomous: correct (= 1) or not (= 0). For the LRMMs, the equation at Level 1 was as follows, where p_*ti*_ is the probability of the dependent variable = 1.

(4) $$\begin{array}{r} \log\left(\frac{p_{ti}}{1-p_{ti}}\right)=\beta_{0i}+\;\beta_{1i}\left(post-test\right)+\;r_{ti}\# \end{array}$$

Level 2 equations were the same as those in the LMM. The LMM and LRMM equatins were generated by S.Y., who was blinded to the allocation of the intervention, while actual analyses were conducted by J.U. The level of significance was set at alpha = 0.05 in all analyses. In addition, a Monte-Carlo-based *post-hoc* power analysis was conducted. Statistical analyses were conducted using R version 3.5.1 with the lmerTest package, lme4 package, and simr package.

### Ethical Aspects and Trial Registration

The project protocol was approved by the human ethics committee of Nara Medical University. This trial was registered in the University Hospital Medical Information Network clinical trials registry (UMIN-CTR; ID = UMIN 000032311; date of registration: April 19, 2018).

## Results

**Figure 1:**
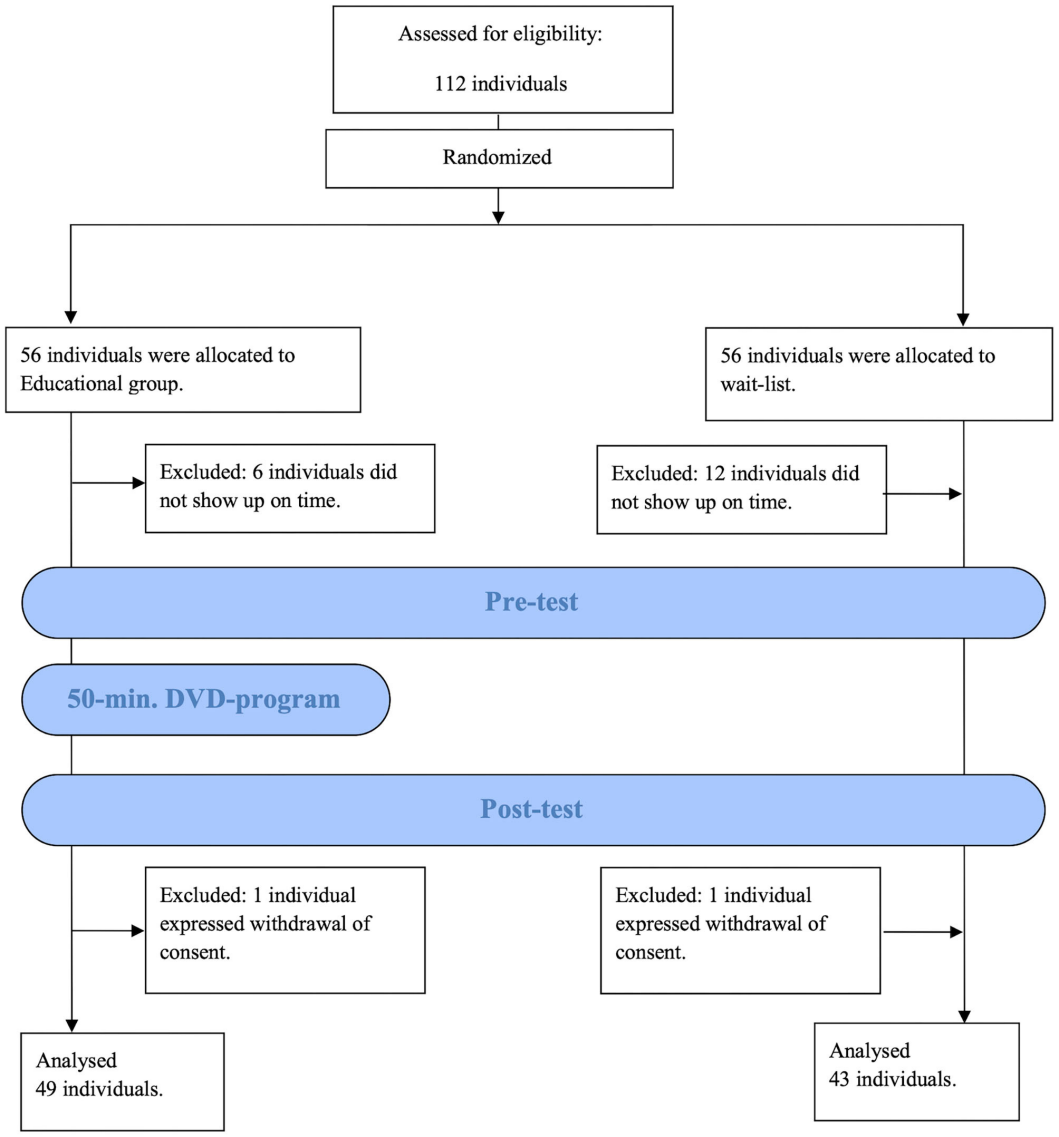
CONSORT flowchart.

We visited and held workshops in a total of four schools. For two schools, we held each educational session in the form of meeting together; for the other two schools, we created a timetable for each so that the teachers' free time could be used and conducted each educational session accordingly. Figure 1 shows the flow of participants at each stage of the trial. We explained the research in advance, obtained consent from a total of 112 schoolteachers, and allocated them to two groups at a ratio of 1:1. Eighteen individuals did not attend as a result of changes to the timetables. Of those who attended, two individuals declined to participate in the research.

Table 4 presents the teachers' demographic information. The sample comprised 92 participants (49 in the educational group and 43 in the control group). The details of the type of school to which each teacher belonged were as follows: 24, elementary schools; 7, secondary schools; 4, special support schools; 30, middle schools; and 27, high schools. Regarding the demographic characteristics (sex, age, and years of work as a teacher), experience of attending seminars regarding mental health, experience of involvement with people with a mental illness, and mental health-related stigma (measured using the past domain of the RIBS-J), the educational group did not differ from the control group. Sixteen individuals with missing data for the RIBS-J were excluded from the analysis of RIBS-J values.

**Table 4:** Demographic characteristics at baseline.

		**Educational group (*n* = 49) Mean (SD)**	**Control group (*n* = 43) Mean (SD)**	***t* or χ^**2**^**	***p***
Age		41.6 (13.5)	40.8 (12.5)	*t*(90) = 0.29	0.77
Gender	Male	28	26	χ^2^ = 0.10	0.83
	Female	21	17	df = 1	
Years of work as teacher		16.5 (12.2)	15.9 (12.4)	*t*(90) = 0.21	0.84
Have you ever attended a seminar about mental health?	Yes	23	23	χ^2^ = 0.39	0.68
	No	26	20	df = 1	
Have you been involved with people with a mental illness?	Yes	30	29	χ^2^ = 0.61	0.51
	No	19	13	df = 1	
RIBS-J past domain ^a)^^,^^b)^		1.1 (1.1)	1.0 (1.1)	*t*(75) = 0.47	0.64

a) * RIBS-J: Japanese version of the Reported and Intended Behavior Scale*;

b) * n (educational group) =39, n (control group) =37*.

The results are presented in **Table 6**. The Monte-Carlo simulation suggested statistical power of ≥0.90 for the current sample sizes (~90), except for domain C, case 2 of domain B, and domain E (Table 5).

**Table 5:** *Post-hoc* power analysis using a Monte-Carlo simulation approach.

		***n* = 20**	***n* = 40**	***n* = 60**	***n* = 80**	***n* = 100**
Domain A		0.99	1.00	1.00	1.00	1.00
Domain B	Q1	0.12	0.25	0.74	0.90	0.97
	Q2	0.07	0.07	0.21	0.37	0.52
	Q3	0.06	0.44	0.75	0.90	0.95
Domain C		0.13	0.18	0.24	0.31	0.35
Domain D		0.77	0.98	1.00	1.00	1.00
Domain E		0.00	0.00	0.00	0.00	0.00

### Domain A. General Knowledge About Mental Health/Illnesses

A significant interaction effect between group and time was observed. In terms of knowledge gained after the intervention, the educational group was shown to have improved to a greater degree than the control group (mean difference = 5.65, 95% CI: 4.54–6.75). The effect size was large (*d* = 1.60) [(30); Table 6].

**Table 6:** Change in each dependent variable (Domain A, B, C, D, E).

			**Domain A**	**Domain C**	**Domain D**	**Domain E**	**Domain B**	**Q1**	**Q2**	**Q3**
Pre	Edu		9.7 (3.42)	12.2 (0.36)	46.8 (7.27)	12.7 (3.16)		51.0	77.6	30.6
	Con	Mean (SD)	10.4 (3.67)	12.8 (0.38)	45.4 (6.47)	13.7 (3.02)	Proportion	44.9	75.5	22.4
Post	Edu		15.6 (2.60)	13.2 (0.36)	53.8 (6.23)	13.5 (3.11)	(%)	89.8	93.9	75.5
	Con		10.6 (3.80)	13.0 (0.38)	45.7 (6.95)	13.2 (3.13)		46.9	73.5	24.5
**Fixed Effects:**			Regression coefficients (95% confidence intervals)				Odds ratio (95% confidence intervals)			
Intercept		γ_00_	10.40^***^	12.77^***^	45.4^***^	13.16^***^	Exp (γ_00_)	1.11	20.85^**^	0.09^**^
			(9.39, 11.40)	(12.02, 13.51)	(43.36, 47.38)	(12.15, 14.17)		(0.18, 6.79)	(4.82, 228.24)	(0.01, 0.40)
Group		γ_01_	−0.68	−0.56	1.44	−0.50	exp (γ_01_)	1.09	0.38	1.92
			(−2.06, 0.70)	(−1.58, 0.46)	(−1.31, 4.19)	(−1.91, 0.91)		(0.10, 13.11)	(0.05, 2.06)	(0.26, 18.10)
Post-test		γ_10_	0.21	0.21	0.37	0.11	Exp (γ_10_)	1.36	0.75	1.31
			(−0.60, 1.02)	(−0.50, 0.92)	(−1.15, 1.90)	(−0.57, 0.78)		(0.29, 7.05)	(0.15, 3.37)	(0.30, 6.17)
Group × Post-test		γ_11_	5.65^***^	0.77	6.67^***^	0.69	Exp (γ_11_)	111.95^**^	11.11^*^	47.88^**^
interaction			(4.54, 6.75)	(−0.21, 1.75)	(4.58, 8.75)	(−0.26, 1.63)		(8.62, 3514.08)	(1.28, 147.31)	(5.30, 813.04)
Cohen's d			1.60	0.30	0.97	0.10		–	–	–
**Random effects:**			Residuals							
Time (Level 1)		var(r_ti_)	3.64	2.86	12.97	2.20		–	–	–
Teacher (Level 2)		var(μ_0i_)	7.79	3.40	32.52	7.68		15.71	4.49	9.57

*Edu, education group; Con, control group; SD, standard deviation*.

* *p < 0.05*;

** *p < 0.01*;

*** *p < 0.001*.

### Domain B. Ability to Recognize Specific Mental Disorders (Depression, Panic Disorder, and Schizophrenia)

The proportion of participants giving correct answers to each question increased (OR = 111.95, 95% CI: 8.62–3514.08 for Q1; 11.11, 95% CI: 1.28–147.31 for Q2; 47.88, 95% CI: 5.30–813.04 for Q3) compared to pre-test in the educational group, significantly more than that in the control group (Table 6).

### Domain C. Depression Stigma Scale

No differences were detected regarding improvements in the DSS scores (mean difference = 0.77, 95% CI: −0.21–1.75). The effect size was small (*d* = 0.30; Table 6).

### Domain D. Intention to Help Students With Depressive Symptoms

Regarding intention to help students, the educational group showed greater improvement in the total score compared to the control group (mean difference = 6.67, 95% CI: 4.58–8.75), compared to pre-test in the educational group, significantly more than the control group. The effect size was large (*d* = 0.97; Table 6).

### Domain E. RIBS-J Future Domain

In terms of decreases in stigma (assessed using the future domain of the RIBS-J), there was no significant difference observed between the educational group and the control group (mean difference = 0.69, 95% CI: −0.26–1.63). The effect size was small (*d* = 0.10; Table 6).

## Discussion

In this two-arm, parallel-group, non-blinded RCT, we tested the effectiveness of a short video-based MHL program that was designed to educate schoolteachers regarding students' mental health and associated stigma. The group who received this educational program was compared with the control group. The teachers who received this program showed greater knowledge gains than the control group. Referring to a systematic review of MHL programs for schoolteachers (24), many studies have reported positive effects on knowledge, and this research corroborates the findings of such studies.

Meanwhile, although this program was not effective for decreasing stigma toward mental illness as measured using the DSS (items 1–3) and the future domain of the RIBS-J, the educational group showed increased intention to assist students with depression. The improvement of knowledge and decreasing of stigmatizing attitudes are not always achieved at the same time [(24), p. 7–13]. Previous findings [(31), p. 170–76] have shown that educating communities about mental health has a relationship with the provision of appropriate help in the future. Therefore, education about MHL might lead teachers to recognize students with mental health problems and provide appropriate help to students. Schools are expected to be effective platforms for both mental health promotion and the implementation of measures to address students' emotional, behavioral, and psychiatric problems.

A key topic of discussion regarding MHL education is how it should be administered and what educational content it should include [(12), p. 154–58]. There is currently no fixed teaching method for MHL. It remains unclear which methods of educating schoolteachers can be expected to most comprehensively improve MHL. According to a previous review [(32), p. 120–33] regarding interventions for reducing stigma, various types of interventions such as contact interventions, lectures, and videos, have been tested in previous research, but it is still not possible to conclude which method is the best. The method implemented in this study ensures uniformity of intervention regardless of the skill of the practitioner and is useful because teachers could take a short video-based MHL program in a short time whenever and wherever they prefer.

Similarly, no conclusions have been reached regarding the optimal teaching content. Continuous acquisition of the latest knowledge concerning screening for and assessing mental illness might involve enormous time and cost. In addition, since the circumstances of mental illnesses vary depending on the country, culture, and era in question, it may be necessary to design a range of programs to suit each environment [(12), p. 154–58].

In Japan, the revised curriculum guidelines require teachers to provide guidance regarding depression, anxiety, schizophrenia, and eating disorders, and it is significant that the teaching materials used in this study included all of these topics. Therefore, it is necessary to continue providing interventions for teachers and to constantly renew the content used to educate schoolteachers.

## Strengths and Limitations

This study has strengths. RCT was selected as the study design, and uniform intervention by DVD was performed. Good results were obtained regarding knowledge provision and behavior prediction. However, several limitations of this study need to be considered. First, the teachers were evaluated immediately before and after the education; however, no long-term follow-up was implemented. Thus, the persistence of the effect of the education could not be determined. Second, this study cannot report whether the program induced a change in teachers' behaviors regarding providing support for their students. Third, as with any self-reported measure, there is the possibility for self-reporting bias. Fourth, the Monte-Carlo simulation revealed some items with insufficient statistical power, raising concern that the sample size was small. This indicates the necessity for increased sample size and future verification of the current results. Finally, we did not examine any associated adverse events, such as whether the lessons affected identification of students with problems and the implementation of treatment interventions for such students.

## Conclusion

This study suggests that a short video-based MHL education can improve schoolteachers' MHL and can increase intention to assist students. This approach affords a wide range of applications and further expansion of the scope in the future. Further research and robust evidence regarding MHL programs' effectiveness in relation to improving mental-health outcomes will be needed to ensure that the best possible education is provided to future generations.

## Data Availability Statement

The raw data supporting the conclusions of this article will be made available by the authors, without undue reservation.

## Ethics Statement

The studies involving human participants were reviewed and approved by The human ethics committee of Nara Medical University. The patients/participants provided their written informed consent to participate in this study.

## Author Contributions

TK designed the study. JU, YM, TM, and KO wrote the protocol and delivered the intervention. TS developed the MHL education DVD. TS, SM, and SY developed the questionnaire. JU undertook the statistical analysis. All authors contributed to and have approved the final manuscript.

## Conflict of Interest

The authors declare that the research was conducted in the absence of any commercial or financial relationships that could be construed as a potential conflict of interest.
